# Does burnout affect clinical reasoning? An observational study among residents in general practice

**DOI:** 10.1186/s12909-020-02457-y

**Published:** 2021-01-07

**Authors:** Philippe Guillou, Thierry Pelaccia, Marie-Frédérique Bacqué, Mathieu Lorenzo

**Affiliations:** 1 Departement of General Practice, Medicine Campus, University of Strasbourg, 4, rue Kirschleger, 67085 Strasbourg Cedex, France; 2 Center for Training and Research in Health Sciences Education, Medicine Campus, University of Strasbourg, 4, rue Kirschleger, 67085 Strasbourg Cedex, France; 3 Prehospital Emergency Care Service, Strasbourg University Hospital, University of Strasbourg, 1, place de l’hôpital, BP 426, 67091 Strasbourg Cedex, France; 4 EA3071, Psychology Faculty, University of Strasbourg, 12, rue Goethe, 67000 Strasbourg, France

**Keywords:** Burnout, Clinical reasoning, General practice, Medical education, Script concordance test

## Abstract

**Background:**

Burnout results from excessive demands at work. Caregivers suffering from burnout show a state of emotional exhaustion, leading them to distance themselves from their patients and to become less efficient in their work. While some studies have shown a negative impact of burnout on physicians’ clinical reasoning, others have failed to demonstrate any such impacts. To better understand the link between clinical reasoning and burnout, we carried out a study looking for an association between burnout and clinical reasoning in a population of general practice residents.

**Methods:**

We conducted a cross-sectional observational study among residents in general practice in 2017 and 2019. Clinical reasoning performance was assessed using a script concordance test (SCT). The Maslach Burnout Inventory for Human Services Survey (MBI-HSS) was used to determine burnout status in both original standards of Maslach’s burnout inventory manual (conventional approach) and when individuals reported high emotional exhaustion in combination with high depersonalization or low personal accomplishment compared to a norm group (“emotional exhaustion +1” approach).

**Results:**

One hundred ninety-nine residents were included. The participants’ mean SCT score was 76.44% (95% CI: 75.77–77.10). In the conventional approach, 126 residents (63.31%) had no burnout, 37 (18.59%) had mild burnout, 23 (11.56%) had moderate burnout, and 13 (6.53%) had severe burnout. In the “exhaustion + 1“ approach, 38 residents had a burnout status (19.10%). We found no significant correlation between burnout status and SCT scores either for conventional or “exhaustion + 1“ approaches.

**Conclusions:**

Our data seem to indicate that burnout status has no significant impact on clinical reasoning. However, one speculation is that SCT mostly examines the clinical reasoning process’s analytical dimension, whereas emotions are conventionally associated with the intuitive dimension. We think future research might aim to explore the impact of burnout on intuitive clinical reasoning processes.

**Supplementary Information:**

The online version contains supplementary material available at 10.1186/s12909-020-02457-y.

## Background

Herbert J. Freudenberger defined burnout as “becoming exhausted by making excessive demands on [ …] resources at work” [1]. The prevalence of burnout among physicians has been reported to be up to 85%, and up to 75% in residents, depending on their medical specialty [2–5]. Burnout has been found, through psychometric tests, to be associated with deteriorations in attention, memory, and executive functions in the general population [6]. The effects of burnout on a physician’s ability to reason have not been extensively studied, and the results are contradictory.

Clinical reasoning encompasses the range of cognitive processes necessary to evaluate and treat patients [7]; it lies “at the core of health care practice and education” [8]. Multiple clinical reasoning components can be identified: information gathering, hypothesis generation, forming a problem representation, generating a differential diagnosis, selecting a leading or working diagnosis, providing diagnostic justification, and developing a management or treatment plan [9]. One of the current main theoretical models assumes the existence of two cognitive processes commonly used by physicians to perform these steps [10, 11]: intuitive processes (system 1) and analytical processes (system 2). Both systems are jointly involved in most physicians’ decisions: reasoning always starts intuitively (system 1), generating one or more possible solutions, and then the analytical system (system 2) will allow confirmation or invalidation of the relevance of these (hypotheses selection) [12].

While some studies have shown a negative impact of burnout on physicians’ clinical reasoning performance [13], others have failed to demonstrate any such impact [14–16]. Residents seemed to be more susceptible to burnout effects than faculty in a study by Durning et al. [13]. Residents had different blood oxygen level-dependent (BOLD) signals detected by functional magnetic resonance when answering and reflecting upon clinical problems [13]. Higher depersonalization scores were associated with a lower BOLD signal in some areas of the brain, and higher emotional exhaustion scores were associated with stronger BOLD signals in others [13].

However, another study on pediatric residents showed no statistically significant association between burnout and harmful, nonharmful, or total errors [14]. A study on internist residents showed no difference in diagnostic and therapeutic accuracy compared with certified internists, despite significantly higher burnout scores [15]. Even more confusing, residents with high burnout scores were reported to have a small decrease in medical errors compared with burnout-free residents in a study on internal medicine residents [16].

To better understand the link between clinical reasoning and burnout, we carried out a study looking for an association between burnout status and lower clinical reasoning performance in a population of residents in general practice.

## Methods

We designed a cross-sectional observational study looking for a statistical association between the scores on the French version of the Maslach Burnout Inventory-Human Services Survey (MBI-HSS) [17] and a script concordance test (SCT) [18]. We assumed that high burnout scores could be associated with a lower SCT rating. We followed the Strengthening The Reporting of OBservational studies in Epidemiology (STROBE) checklist on what should be included in an accurate and complete report of an observational study [19].

### Setting

We collected data on residents’ certification examinations in 2017 and 2019 at the University of Strasbourg, France. Participants were given a presentation of the study, a written consent form, and the MBI-HSS questionnaire. We informed the residents of the research goal orally and ensured the voluntary nature of their participation before the start of the examination. The SCT examination lasted 90 min, and the residents had to stay until the end. Two of the researchers (PG and ML) were faculty for the residents.

### Population

The study population consisted of general practice residents in their final year (third postgraduate year) at the University of Strasbourg. To participate, residents had to give written informed consent. Participation or nonparticipation did not influence residents’ training programs or assessments.

As participants could realize that they suffered from burnout, they received the contact details of adequate support resources with the consent form before the study.

### Variables under consideration

We collected information on sex, age, marital and parental status, and current residency workplace, as these variables might influence burnout and clinical reasoning, as shown in previous studies [13, 20].

#### Burnout

The MBI-HSS is the most commonly used tool to assess burnout in the medical population [5, 21]. It is a validated self-report questionnaire measuring the three dimensions of burnout: emotional exhaustion, depersonalization, and reduced personal accomplishment [22]. The MBI-HSS gives a score in each of burnout’s three dimensions. Residents were asked to indicate, on a Likert scale, their degree of agreement with statements on a 9-item emotional exhaustion scale (e.g., “I feel I’m working too hard at my job”), a 5-item depersonalization scale (e.g., “I do not truly care what happens to some patients”), and an 8-item personal accomplishment scale (e.g., “I have accomplished many worthwhile things in this job”). Each item scores from 0 to 6 on a Likert frequency scale: 0 = never, 1 = at least a few times a year, 2 = at least once a month, 3 = a few times a month, 4 = once a week, 5 = a few times a week, and 6 = every day. We used the French version of the MBI-HSS from Mind Garden, Inc., with a license to reproduce.

We chose to use two of the main burnout cutoffs described in the literature, as there is an intense debate on the best way to define them [5, 21, 23]. No single method seems to enjoy a consensus.

First, burnout score severity cutoffs in this study were based on the original standards of Maslach’s burnout inventory manual, revised in this French population [3, 17, 22, 24] (see Table 1). We will refer later to this method as the “conventional approach.” Defining each dimension’s cutoff remains controversial, as they may differ from one population to another [5, 21, 25, 26]. We chose these cutoffs, as they were used 10 years ago in a similar population to evaluate burnout [3].

**Table 1:** MBI-HSS burnout dimension cut-offs in the conventional approach

	Low	Moderate	High
Emotional exhaustion	≤17	18–29	≥30
Depersonalization	≤5	06–11	≥12
Personal accomplishment	≥40	34–39	≤33

The number of highly affected dimensions defined burnout: low with a high score in only one dimension, moderate with high scores in two dimensions, and high with high scores in all three dimensions.

Second, we analyzed the overall burnout level using the “exhaustion + 1” rule applied to MBI-HSS scores: individuals can be considered burned out when, compared to a norm group, they report high emotional exhaustion in combination with high depersonalization or low personal accomplishment [23]. In this approach, “high” means scoring in the 75th percentile or higher, while “low” refers to scoring in the 25th percentile or lower [23]. We used this method to determine burnout cutoffs, as it considers burnout more as a continuum than a predefined cutoff [21].

#### Clinical reasoning

Evaluation of the clinical reasoning process is a complicated task with no ideal single assessment tool [27]. Some authors consider that testing clinical reasoning in the context of uncertainty and respecting the possibility of more than one good option are two core principles [28]. The script concordance test (SCT) is currently one of the most powerful tools available to assess clinical reasoning under these principles [28]. SCTs are meant to measure the degree of concordance between examinees and a reference panel of experts concerning clinical decisions and actions under uncertainty. For each item, a clinical case is presented (a vignette), containing either insufficient information to solve the clinical problem (diagnostic, treatment) or ambiguous data. A series of questions is related to the case. Each contains an option relevant to the clinical problem, followed by the presentation of new information. The examinees’ task is to assess the effect this new information has on the option’s status. It mostly examines the hypothesis selection stage in the clinical reasoning process [27, 29]. It is thought to explore system 2 rather than system 1 [18]. SCT is used in many curricula worldwide to evaluate clinical reasoning among pre- and postgraduate medical students.

We used SCT scores to evaluate clinical reasoning in our population. We created a 90-item SCT on general practice [30]. We used Lubarsky et al.’s guide to develop this SCT [31] and recommendations from Dory et al. to recruit panel members and analyze scores [32]. Scores are expressed as percentages for the analysis. A pass cutoff at 60% is usually recommended. This passing cutoff is determined by the mean score of panel members (usually approximately 80%). A score < 60% is then considered too far from what is the “good” answer to be accepted.

We chose to use universal anchors to be able to mix every type of SCT question in each vignette when wanted. One question from this SCT is presented as an example in Table 2.

**Table 2:**
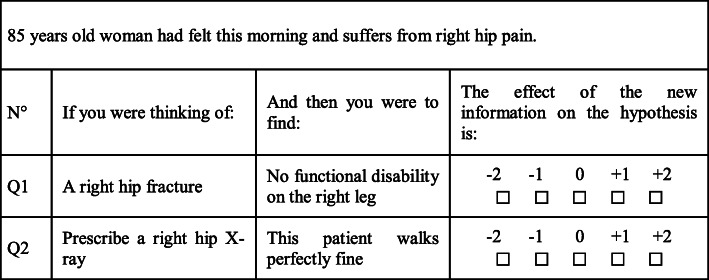
Example of SCT questions

−2: strongly negative; − 1: negative; 0: no effect; + 1: positive; + 2: strongly positive

### Statistical analysis

For 80% power and an alpha risk of 5%, 20 or more participants from each group (with or without burnout) were required to show a difference of 1 point or more in SCT scores.

The sample was subjected to descriptive statistical analysis. Means are presented in the results with 95% confidence intervals and the standard deviation or the minimum and maximum values.

The mean SCT scores were compared for qualitative variables of sex, marital status, and parental status using the Mann-Whitney test. We studied the statistical association between the SCT results and the quantitative age variables and the different MBI scores for each burnout dimension by calculating the Pearson correlation coefficients (Rho). Finally, we compared the means of the SCT score in the subgroups classified according to burnout severity using the Kruskal-Wallis test for the conventional burnout method and Student’s t-test for the burnout “exhaustion + 1” method. Statistical analysis was performed using R through the GMRC Shiny Stats application and RStudio version 1.2.1335.

## Results

### Population

In 2017, 111 (86.7%) residents agreed to participate. In 2019, 88 (66.7%) residents agreed, for a total of 199 participants. Informed consent was obtained for all participants.

Almost two-thirds of the participants were women (*n* = 123). Most of the participants were in a relationship (*n* = 139; 83.2%; 32 missing data) and had no children (*n* = 113; 86.3%; 68 missing data). The participants’ average age was 28 [min-max: 26–42; two missing data].

### SCT scores

The mean SCT score of our participants was 76.44% (95% CI: 75.77–77.10). The minimum score was 61.30%, and the maximum score was 88.96% (SD = 4.78).

The global SCT score of all residents for these 2 years (*n* = 259) was 76.41% (95% CI: 75.77–77.04). There was no significant difference between the global mean scores of all residents and participants (*p* = 0.4738).

### Burnout assessment

In the conventional burnout cutoff approach, 126 residents (63.31%) had no burnout, 37 (18.59%) had mild burnout, 23 (11.56%) had moderate burnout, and 13 (6.53%) had severe burnout. The mean scores were 20.88 for emotional exhaustion, 9.57 for depersonalization, and 38.52 for personal accomplishment.

Table 3 illustrates the distribution of burnout dimension scores in the conventional cutoff approach.

**Table 3:** Repartition of burnout dimension scores in the conventional approach

	Low score	Moderate score	High score
Emotional exhaustion	82 (41.21%)	73 (36.68%)	44 (22.11%)
Depersonalization	57 (28.64%)	70 (35.18%)	82 (41.21%)
Personal accomplishment	94 (47.24%)	69 (69.70%)	36 (18.09%)

In the “exhaustion + 1” approach, 38 residents had a burnout status (19.10%). High and low cutoffs for burnout dimension scores are presented in Table 4.

**Table 4:** MBI-HSS burnout dimensions cut-offs in the “exhaustion +1” approach

	Low score	Moderate score	High score
Emotional exhaustion	≤12	13–27	≥28
Depersonalization	≤5	5–12	≥13
Personal accomplishment	≥43	34–42	≤35

### SCT and MBI score association

There was no statistically significant correlation between SCT scores and burnout status in the conventional cutoff approach (*p* = 0.6509). Details of SCT scores concerning burnout severity are presented in Table 5.

**Table 5:** Comparison of SCT mean scores on burnout severity in the conventional approach

	Means SCT score (95%CI)	*p*-value
Burnout absence	73.29% (72.32–74.29)	*p* = 0.6509
Mild burnout	71.45% (69.08–73.83)	
Moderate burnout	74.96% (72.43–77.49)	
Severe burnout	71.83 (70.31–73.36)	

There was also no statistically significant difference between SCT scores and burnout status in the “exhaustion + 1” approach: mean SCT with burnout was 73.48% [95% CI: 71.76–75.21] versus 72.95% [95% CI: 72.01–73.88] without burnout; *p* = 0.6136.

## Discussion

### Comparison with the literature

In the conventional approach, the mean burnout scores found in our study in each dimension were slightly lower than those found in a recent a literature review conducted by Erschens et al. among medical residents for emotional exhaustion (20.58 versus 22.9, respectively) and personal accomplishment (38.53 versus 35.1, respectively). However, it was higher for depersonalization (9.57 versus 8.9, respectively) [5]. Similarly, our mean burnout scores were comparable with those of a national study in France among general practice residents from 2011: 20.0 for emotional exhaustion, 9.7 for depersonalization, and 34.8 for personal accomplishment [3]. The prevalence of burnout was lower in our population than in the authors’ study: 36.68% versus 48.1% [3]. We found fewer residents with burnout in the conventional approach than in a study from 2009 at the Strasbourg medical school (46%) [24]. Our resident population seemed to suffer less from burnout than other studies.

SCT scores were comparable with the values expected for such a postgraduate examination [32]. The mean SCT score for residents at the end of their formation is 75% [32].

Our results contradict our initial hypothesis, assuming that high burnout scores could be associated with a lower SCT rating. There seems to be no significant association between clinical reasoning measured by SCT and burnout within our experimental conditions.

Therefore, our results are in line with several studies that showed no negative effect of burnout on clinical reasoning in various settings [14–16]. Several factors could explain these results. In the “emotional exhaustion +1” approach, burnout is an emotional syndrome. As emotions are known to impact clinical reasoning [33, 34], some authors argue that emotions have a greater impact on the system 1 reasoning process [35]. An SCT-specific reasoning task consisting of analyzing the impact of information on a hypothesis or an investigation option is a hypothetical-deductive process that fosters and explores system 2 [8, 11]. Consequently, one speculation regarding our results is that burnout might not affect system 2.

However, no specific data on clinical reasoning behaviors with SCT are available. One study by Surry et al. examined clinical reasoning behaviors in a 210-item clinical-vignette MCQ test based on dual-process theory [36]. The results showed that both systems 1 and 2 processes were elicited for nearly all test questions (100 and 97.1%, respectively) in a small sample of subjects [36]. Further studies are needed to explore system 1 and system 2 reasoning use during an SCT to support the assumption that SCT mostly explores system 2. Finally, our findings illustrate some of the difficulties in studying the links between clinical reasoning and burnout.

Another hypothesis would be that burnout does not affect clinical reasoning at all. Considering this would question the affective valence of intuitive reasoning. Many authors, such as Croskerry, have argued for over a decade that emotions do impact clinical reasoning [10, 37]. As such, other studies might aim to specifically explore the impact of burnout on clinical reasoning system 1 to test the impact of burnout on clinical reasoning more specifically.

### Strengths and weaknesses

Our study’s main strength is its originality, as few data on the links between burnout and clinical reasoning performance are available. To the best of our knowledge, no other studies used SCT to study the links between burnout and clinical reasoning.

The use of a standardized and validated burnout questionnaire given to the whole study population has reduced subjectivity bias. The oral and written study presentation included no hypothesis but rather a broad research question to avoid influencing the participants’ answers to the MBI-HSS questionnaire.

Burnout is impacted by the environment and context of the individual. We assume that being out of the real professional environment in a quiet classroom may have reduced tension for some residents. Clinical reasoning in an SCT question probably does not imply as much emotion as with a real patient. This could be another limitation of our findings. Having the subject perform a clinical reasoning task under more realistic workplace conditions could improve our research’s validity.

We noticed a slight decrease in SCT scores with increased burnout levels. There might be an association that is not reflected in our study because it lacks power. A larger sample size could probably show a statistically significant difference between the groups. However, even if such a difference existed, the estimated effect size would be much lower than a standard deviation.

Measuring burnout remains challenging today. No assessment tool seems ideal, and there is much debate on the best definition of burnout [21]. The heterogeneity of research on burnout is called into question, as no less than 142 unique definitions of burnout were found in a recent review [21]. Thus, the clinical validity of burnout definitions are not certain [21]. The fourth edition of the MBI manual removed the cutoffs [38] of the classical approach and preconized the calculation of individuals’ latent profiles for burnout [39]. These latent burnout profiles could be used for future research.

Likewise, assessing clinical reasoning with a single tool such as SCT may not be sufficiently valid for such a complex process [27]. Future studies on clinical reasoning and burnout may use a wide range of assessment methods, such as SCT + OSCE + direct observations + global assessments + think-aloud techniques [27].

Last, it is unclear whether nonparticipants and participants in the studied population are equivalent concerning burnout.

## Conclusions

Our data with SCT scores seem to indicate that burnout status has no significant impact on clinical reasoning among GP residents. However, burnout may either truly have no impact on clinical reasoning performance or impact system 1 processes. Overall, researching the links between burnout and clinical reasoning is complex. Further studies could explore such an impact of burnout on clinical reasoning system 1, but this will not be an easy task. To our knowledge, there is no valid easy method capable of separating these two closely related processes. Mixed methods approaches, where qualitative studies are integrated with quantitative studies, might be a fruitful avenue for future research.

## Supplementary Information

**Additional file 1.** All data of participants including MBI-HSS responses, SCT scores, and descriptive statistical analysis.

## Data Availability

The dataset supporting the conclusions of this article is included in the article in Additional file 1.
